# SaO_2_/FiO_2 _ratio as risk stratification for patients with sepsis

**DOI:** 10.1186/cc12951

**Published:** 2013-11-05

**Authors:** Adriell Ramalho Santana, Jaqueline Lima de Sousa, Fábio Ferreira Amorim, Bárbara Magalhães Menezes, Fernanda Vilas Bôas Araújo, Felipe Bozi Soares, Louise Cristhine de Carvalho Santos, Mariana Pinheiro Barbosa de Araújo, Pedro Henrique Gomes Rocha, Pedro Nery Ferreira Júnior, Alessandra Vasconcelos da Silva Paiva, Gabriel Kanhouche, Alethea Patrícia Pontes Amorim, José Aires de Araújo Neto, Edmilson Bastos de Moura, Marcelo de Oliveira Maia

**Affiliations:** 1 Escola Superior de Ciências da Saúde, Brasília, Brazil; 2 Liga Acadêmica de Medicina Intensiva de Brasília, Brazil; 3 Hospital Santa Luzia, Brasília, Brazil

## Background

The PaO_2_/FiO_2 _ratio is a well-known parameter to assess respiratory dysfunction, used in Sequential Organ Failure Assessment (SOFA) [1]. This study aims to determine whether the SaO_2_/FiO_2 _ratio can be used in the assessment of respiratory impairment and as a predictor of ICU mortality in patients with sepsis and to evaluate its correlation with PaO_2_/FiO_2_.

## Materials and methods

A retrospective cohort study conducted in the ICU of Hospital Santa Luzia, Brasilia, DF, Brazil, during 5 months. An arterial blood sample was collected at the time of admission. Patients with sepsis were divided into two groups: survivors group (SG) and nonsurvivors group (NSG). Correlation with SaO_2_/FiO_2 _and PaO_2_/FiO_2 _was evaluated with the Pearson correlation coefficient. Accuracy of SaO_2_/FiO_2 _and PaO_2_/FiO_2 _to predict ICU mortality was measured with the area under the receiver operating characteristic curve.

## Results

**Figure 1:**
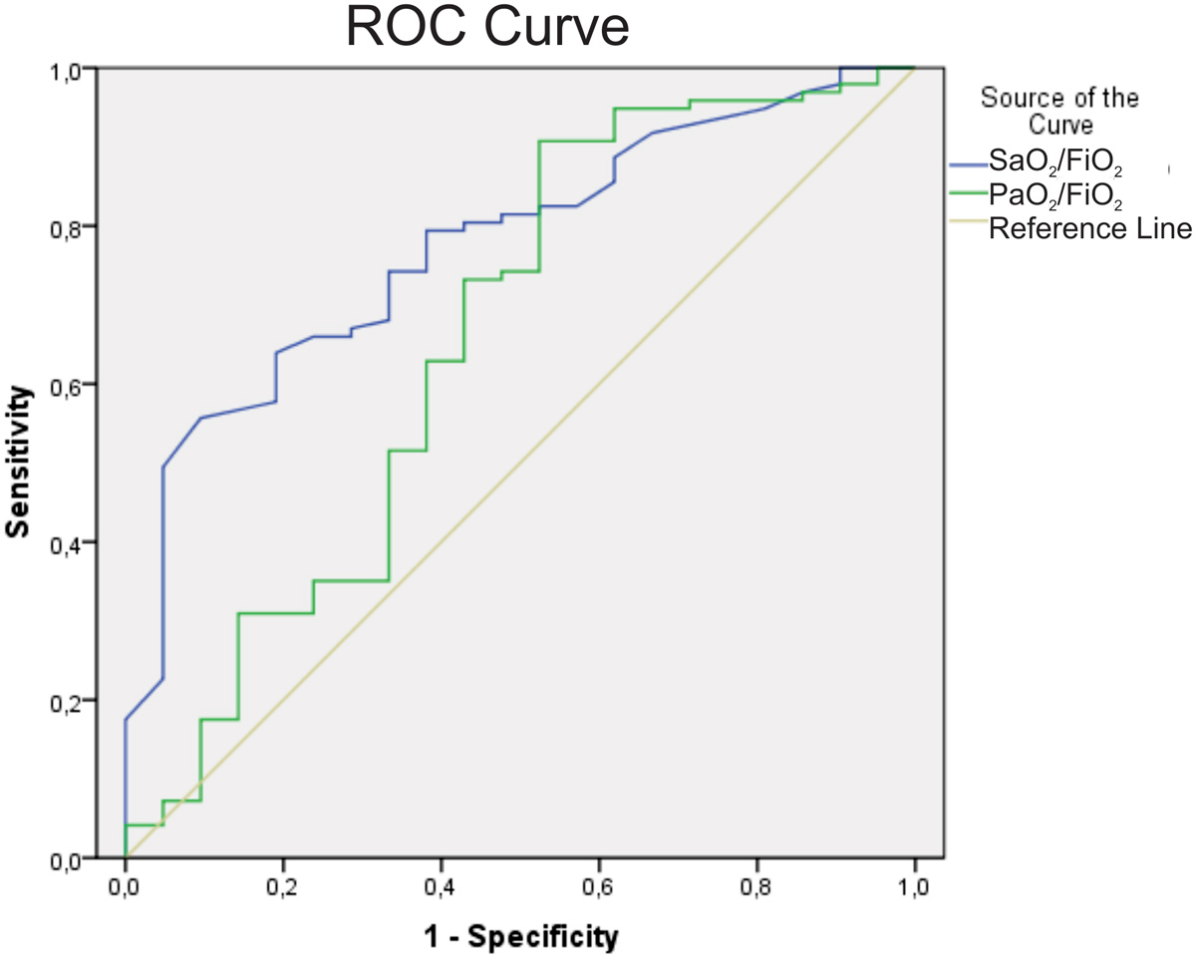
ROC curve for SaO_2_/FiO_2 _and PaO_2_/FiO_2_.

A total of 118 patients with sepsis were enrolled. The mean age was 66 ± 21 years, SAPS3: 50 ± 14, APACHE II: 13 ± 8, PaO_2_/FiO_2_: 317 (IQ 233 to 426) and SaO_2_/FiO_2_: 362 (IQ 247 to 453). ICU mortality was 17.8% (*n *= 21). The main sites of infections were respiratory (57%, *n *= 67), urinary (19%, *n *= 23) and cutaneous (8.5%, *n *= 10). Nonsurvivor patients had lower SaO_2_/FiO_2 _(258 vs. 366, *P *= 0.00) and PaO_2_/FiO_2 _(285 vs. 354, *P *= 0.04). PaO_2_/FiO_2 _and SaO_2_/FiO_2 _had a good correlation (*r *= 0.645, *P *= 0.00). The relative risk of death in patients with SaO_2_/FiO_2 _<400 was 1.81 (95% CI: 1.47 to 2.24), SaO_2_/FiO_2 _<300 was 2.5 (95% CI: 1.54 to 4.05), SaO_2_/FiO_2 _<200 was 2.45 (95% CI: 1.27 to 4.71). The sensitivity for ICU mortality of SaO_2_/FiO_2 _<300 was 28% and of SaO_2_/FiO_2 _<200 was 35%. The specificity for ICU mortality of SaO_2_/FiO_2 _<300 was 90% and of SaO_2_/FiO_2 _<200 was 86% (95% CI: 93.5 to 100.0%). The area under the ROC curve for SaO_2_/FiO_2 _was 0.776 (95% CI: 0.677 to 0.875) and for PaO_2_/FiO_2 _was 0.655 (95% CI: 0.507 to 0.804) (Figure 1).

## Conclusions

A low SaO_2_/FiO_2 _was associated with mortality in this group of patients and had a good correlation with PaO_2_/FiO_2_. SaO_2_/FiO_2 _<300 showed high specificity for mortality. Further analysis is necessary to the validation of less invasive measures such as pulse oximetry saturation (SpO_2_/FiO_2 _ratio) in the assessment of patients with sepsis.
